# Epidural Analgesia in a Parturient for Labor Analgesia After Surgical Correction of Spina Bifida

**DOI:** 10.7759/cureus.69676

**Published:** 2024-09-18

**Authors:** Nicholas R Washburn, Emily Smith

**Affiliations:** 1 Anesthesiology, Kaweah Delta Health Care District, Visalia, USA; 2 Anesthesiology, Kaweah Health Medical Center, Visalia, USA

**Keywords:** anesthesiology, epidural analgesia, epidural anesthesia, labor analgesia, spina bifida, spine surgery

## Abstract

This case report details the successful management of labor pain using epidural analgesia in a 22-year-old primigravid patient with a history of spina bifida and extensive surgical repair. Spina bifida, a congenital neural tube defect, presents unique challenges for neuraxial anesthesia due to altered spinal anatomy and potential neurological deficits. Despite these complexities, effective pain relief was achieved through careful patient counseling, comprehensive pre-procedural planning, and meticulous technique. Upon admission for labor induction due to preeclampsia, a physical examination revealed significant lumbar scarring with obscure anatomical landmarks. Despite these challenges, informed consent was obtained, and an epidural catheter was successfully placed using palpable bony prominences above the presumed T12-L1 interspace. A standard midline approach with saline loss of resistance technique was employed, epidural space was confirmed at a depth of 5 cm, and a catheter was placed. Continuous patient-controlled epidural analgesia (PCEA) comprising fentanyl and ropivacaine provided consistent pain relief throughout a 17-hour labor period, maintaining a dermatome level of T10. Post-delivery, neurosurgical evaluation confirmed successful catheter removal without complications, and the patient exhibited normal neurological function. This case underscores the importance of individualized pain management strategies in patients with spina bifida, necessitating thorough risk-benefit assessment, detailed patient education, and interdisciplinary collaboration for optimal outcomes. Despite the inherent challenges, epidural analgesia can be a viable option with careful planning and execution, contributing to improved maternal comfort and satisfaction during labor and delivery for patients with a history of spina bifida.

## Introduction

This case report discusses the successful management of labor pain in a patient with a history of extensive surgical repair of spina bifida using epidural analgesia. Spina bifida is a congenital neural tube defect that can lead to various neurological and musculoskeletal complications. Effective neuraxial analgesia in such patients can be challenging due to altered anatomical and physiological considerations. In these circumstances, it is common that the spinous process of multiple vertebrae, which are classic landmarks for the guidance of placement of an epidural catheter, is significantly altered or absent. Even if these landmarks are patent, the underlying epidural space may be completely obliterated. This case highlights the importance of including the patient in pain management planning, taking a thorough patient history, and weighing the risks and benefits of treatment options to achieve optimal outcomes.

Spina bifida is characterized by congenital incomplete closure of the neural tube during embryonic development. This process can result in a wide range of abnormalities, including neurological and orthopedic problems such as motor deficits, musculoskeletal defects, and sensory disturbances. Neuraxial techniques for the management of labor pain in these patients pose a particular challenge due to a multitude of possible complicating factors, including altered internal spinal anatomy, missing anatomic landmarks, pre-existing neurologic deficits, and post-surgical changes, amongst others. Epidural analgesia is a well-established technique for providing adequate relief of typical labor pains. When patients with a prior diagnosis of spina bifida are identified, anesthesiologists must carefully assess whether this modality is appropriate or feasible and have a frank and truthful discussion with the parturient. Risks associated with this technique, including inadvertent dural puncture, no relief or “patchy” relief, as well as risks of worsening neurologic deficits, must be discussed. The totality of these factors, both from the standpoint of risks associated with difficult placement and possible failure, epidural analgesia, remains a controversial topic. Despite these challenges, this case demonstrates that a successful labor epidural without complications is possible using a cautious technique combined with a thorough discussion and an eager patient.

## Case presentation

A 22-year-old, G2, term 0, preterm 0, abortion 1, living 0, 97 kg female with a history of spina bifida, extensive surgical repair in infancy, and subsequent tethered cord syndrome requested epidural analgesia for labor analgesia. There was no prior history of neuraxial anesthesia. This term singleton pregnancy was complicated by preeclampsia leading to induction of labor; she was experiencing irregular contractions upon admission. Upon initial presentation, no imaging was available. A physical exam revealed extensive scarring with no palpable landmarks in the lumbar region. A bony process presumed to be a spinous process was palpable at an estimated T12 level. Neurological exam and history revealed no sensory or motor deficits.

The patient was counseled that epidural catheter placement carries a significantly greater risk for the spina bifida patient population. Additional or increased risks discussed were failure to access the epidural space, inadvertent dural picture with accompanying headache, incomplete or “patchy” pain relief, high epidural blockade, nerve damage, and increased length of time for placement. The patient acknowledged the risks, displayed full understanding, and elected to proceed.

**Figure 1:**
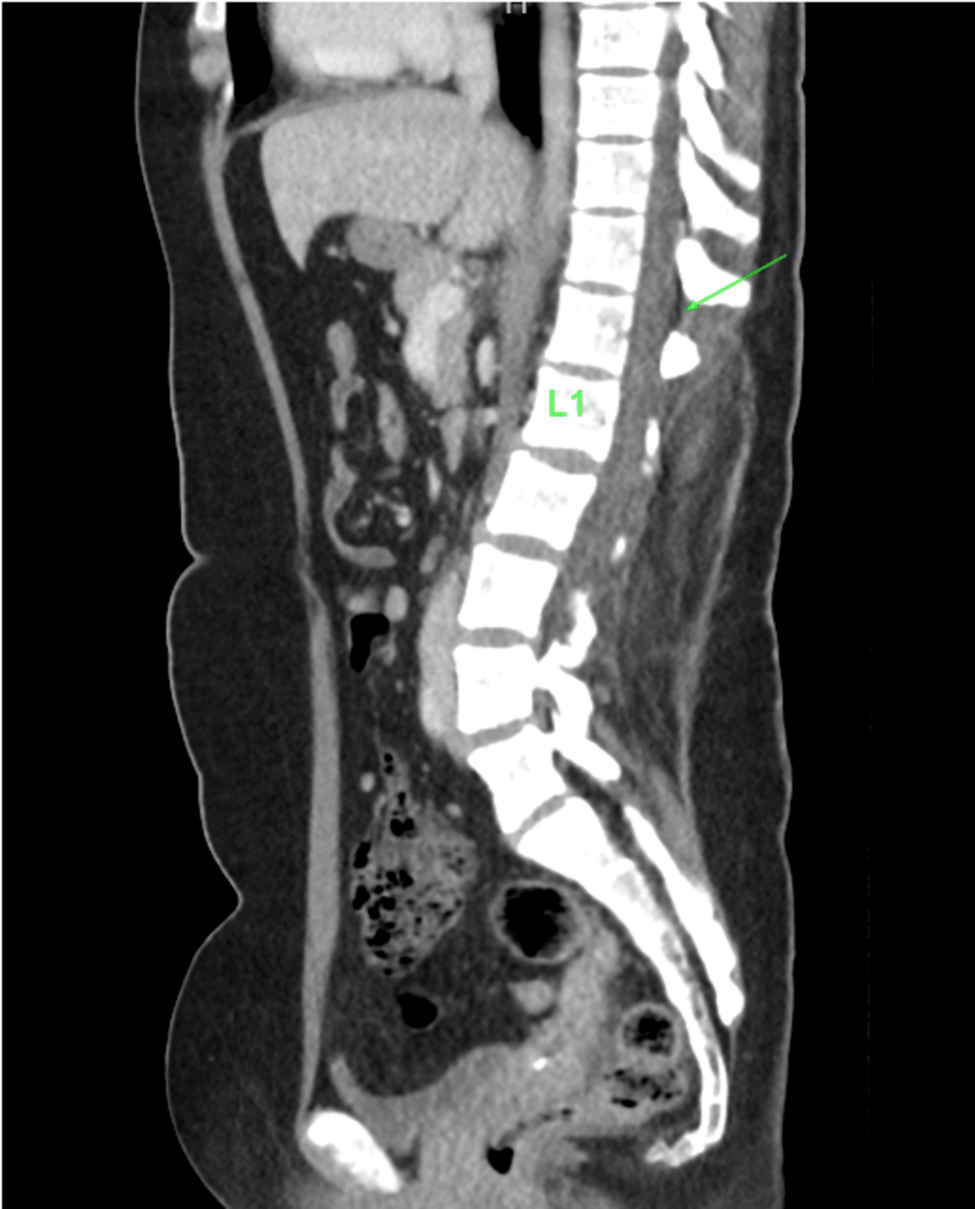
Sagittal computerized tomography scan of abdomen, arrow indicates possible epidural space

**Figure 2:**
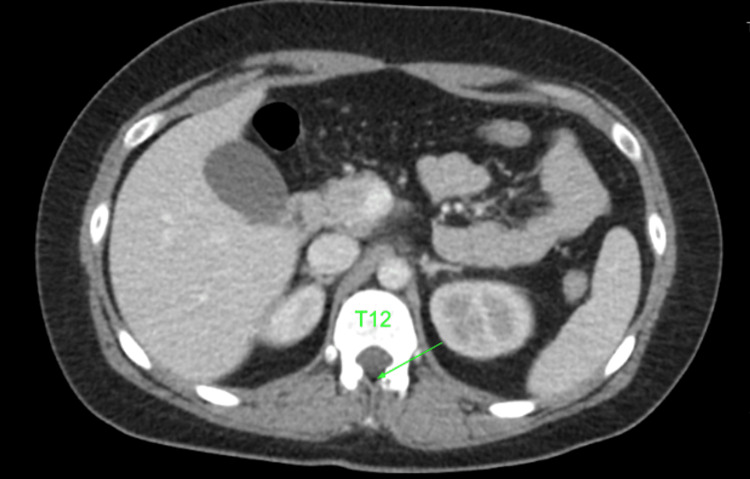
Transverse computerized tomography scan of abdomen, arrow indicates potential epidural space

After palpation of remaining anatomic landmarks, the decision was made to proceed with epidural catheter placement just above the level of the surgical scar and at the first palpable bony prominence, which was presumed to be the T12-L1 interspace. The patient was placed in the sitting position, and the procedure was performed with standard sterile precautions, a 17 g Tuohy needle, and a 19 g epidural catheter. From a midline approach, the loss of resistance to saline technique was utilized. The epidural space was accessed at a depth of 5 cm, and 3 cm of the catheter was left in the epidural space via the Touhy needle. A test dose was administered through the catheter of 3 mL of 1.5% lidocaine and 1:200,000 epinephrine. Following a negative test dose, patient-controlled epidural analgesia (PCEA) dosing was initiated using a solution containing fentanyl 2 mcg/mL and ropivacaine 0.125% at a basal rate of 8 mL/hour with a PCEA bolus of 3 mL and interval lockout of 15 minutes and a one-hour dose limit of 30 mL. No bolus was given due to the perceived risk of a high epidural. Patients, family members, and nursing staff received extensive education on the signs and symptoms of a high epidural blockade. The pain was well controlled with a dermatome level of T10 throughout labor, and vaginal delivery was completed after approximately 17 hours. The epidural catheter was removed with the tip intact without complications. A prior computed tomography scan of the abdomen was discovered after our procedure. Upon review, the epidural space appears to be completely scarred below the T12-L1 interspace. A small patent epidural space can be identified in Figures 1-2 at the T11-T12 interspace.

Effective pain management was achieved throughout labor. On postpartum day 1, the patient was found to have normal neurologic function marked by normal ambulation and return of bowel and urinary function. The patient denied headaches or any signs and symptoms of secondary complications of epidural catheter placement.

## Discussion

Spina bifida occulta may be present in up to 5-10% of the population [1,2]. Spina bifida patients are more likely to require a cesarean section, and traditionally, neuraxial techniques have been avoided due to concerns of secondary complications [3]. Additionally, previous spinal surgery has been considered a relative contraindication to the use of neuraxial anesthetic techniques. The anatomy of the epidural space at the level of the spinal lesion following surgical correction remains unclear. It has been described as "lobules of fat lying in the midline adherent to fascia and surrounding tissues or entirely obliterated" [4,5]. An attempt at epidural placement at the level of the defect significantly increases the risk of incidental dural puncture. A blood patch as a treatment for an inadvertent dural puncture presents similar technical difficulties and may be ineffective. Even if neuraxial needle placement is successful, then the spread of local anesthetic is rather unpredictable and may produce an incomplete block. Despite this, multiple case reports exist in the literature describing the successful use of epidural analgesia in patients with spina bifida both with and without corrective surgery [6,7]. There are no definitive guidelines in place for epidural analgesia in spina bifida patients after spine surgery.

Daley et al. [8] reviewed the experience of 18 patients for a total of 21 separate attempts at epidural analgesia for obstetric patients with previous spinal surgery. Continuous labor analgesia was established in 20 out of 21 cases reviewed. However, only 10 of them were placed easily on the first attempt, leaving the remaining 11 with higher requirements of local anesthetic and/or an incomplete block. Hubbert [9] attempted epidural analgesia in 17 laboring patients with a history of Harrington rod fusion and was successful in nine. It is noted that in only four (23%) was epidural analgesia easily obtained, two patients had inadvertent spinal anesthesia from a dural puncture, and 11 required multiple attempts at various levels. Tidmarsh [10] attempted 10 epidural catheter placements for labor analgesia in a parturient with known neural tube defects, of which four had prior corrective surgery. A similar approach was taken as in our case with a midline approach just above the level of defect at first palpable normal spinous process. Out of the 10 epidurals placed, six received normal symmetrical block at or below low thoracic dermatome levels. Of the four remaining, one block was asymmetric, one excessively high blockade, and two were inadequate analgesia below the level of spinal defect. Notably, of the four epidurals placed in patients with a history of corrective surgery, two received normal sensory blockade, one had an excessively high level (T3) of block, and one had inadequate analgesia at a level below the defect. Therefore, in this case series, 50% of those with corrective surgery had complications or inadequate labor analgesia.

It is worth discussing alternative techniques used to place epidural catheters, which include fluoroscopic and ultrasound-guided techniques. Fluoroscopic guided technique has been shown to have a "higher success rate than traditional landmark technique as it can accurately identify anatomical structures and epidural spaces using imaging devices" [11]. However, due to the difficulty of using fluoroscopy in daily practice and the burden of radiation exposure, the practical use is rather limited, especially in the obstetric population. The role of ultrasound guidance in placing epidural catheters is relatively new, but the evidence regarding its practical application and efficacy continues to grow. Chin et al. performed a thorough literature review regarding the benefits and limitations of ultrasound-guided thoracic and lumbar neuraxial blockades. They discovered the evidence suggests ultrasound guidance could improve neuraxial success rate, quality of epidural analgesia, ease of performance, identification of required needle depth, and identification of intervertebral levels [12]. However, the ultrasound-guided technique has displayed limitations in obese and elderly patients, and its use in those with prior spinal surgery remains unknown [12]. Also, extensive experience with ultrasound-guided techniques may be required before competence is attained [12]. In this case, an anesthesiologist with this experience was not readily available.

Our case is unique in that inadequate imaging was available prior to the placement of the epidural catheter and using a surgical scar as a landmark for a potentially patent epidural space proved to be a successful approach. Additionally, it is unusual given the extent of her post-surgical scarring that a continuous epidural space would retain the patency necessary for successful labor analgesia to be achieved.

## Conclusions

Effective pain management for patients with a history of spina bifida requires a personalized approach due to their unique anatomic and physiologic factors. A clear discussion about the potential complications and the challenges of effective pain control even with safe epidural placement must be had with this patient population. Despite these challenges, epidural catheter placement can be a safe and effective means of achieving labor analgesia. Collaborating with specialists and considering various imaging modalities may enhance the precision of epidural placement and improve overall outcomes.
